# Motion artifacts and image quality in stroke MRI: associated factors and impact on AI and human diagnostic accuracy

**DOI:** 10.1007/s00330-025-11807-7

**Published:** 2025-07-15

**Authors:** Christian Hedeager Krag, Felix Christoph Müller, Karen Lind Gandrup, Michael Brun Andersen, Jakob Møllenbach Møller, Marie Louise Liu, Anita Rud, Simon Krabbe, Lamaa Al-Farra, Mads Nielsen, Christina Kruuse, Mikael Ploug Boesen

**Affiliations:** 1 Department of Radiology, University Hospital Copenhagen-Herlev and Gentofte, Copenhagen, Denmark; 2 Faculty of Health and Medical Sciences, University of Copenhagen, Copenhagen, Denmark; 3 Radiology AI Testcenter (RAIT.dk), Copenhagen, Denmark; 4 Department of Computer Science, University of Copenhagen, Copenhagen, Denmark; 5 Department of Brain and Spinal Cord Injury, University Hospital Copenhagen–Rigshospitalet, Copenhagen, Denmark; 6 Department of Neurology, University Hospital Copenhagen-Herlev and Gentofte, Copenhagen, Denmark; 7 Department of Radiology, University Hospital Copenhagen-Bispebjerg and Frederiksberg, Copenhagen, Denmark

**Keywords:** Brain, Stroke, Magnetic resonance imaging, Artifacts, Artificial intelligence

## Abstract

**Objectives:**

To assess the prevalence of motion artifacts and the factors associated with them in a cohort of suspected stroke patients, and to determine their impact on diagnostic accuracy for both AI and radiologists.

**Materials and methods:**

This retrospective cross-sectional study included brain MRI scans of consecutive adult suspected stroke patients from a non-comprehensive Danish stroke center between January and April 2020. An expert neuroradiologist identified acute ischemic, hemorrhagic, and space-occupying lesions as references. Two blinded radiology residents rated MRI image quality and motion artifacts. The diagnostic accuracy of a CE-marked deep learning tool was compared to that of radiology reports. Multivariate analysis examined associations between patient characteristics and motion artifacts.

**Results:**

775 patients (68 years ± 16, 420 female) were included. Acute ischemic, hemorrhagic, and space-occupying lesions were found in 216 (27.9%), 12 (1.5%), and 20 (2.6%). Motion artifacts were present in 57 (7.4%). Increasing age (OR per decade, 1.60; 95% CI: 1.26, 2.09; *p* < 0.001) and limb motor symptoms (OR, 2.36; 95% CI: 1.32, 4.20; *p* = 0.003) were independently associated with motion artifacts in multivariate analysis. Motion artifacts significantly reduced the accuracy of detecting hemorrhage. This reduction was greater for the AI tool (from 88 to 67%; *p* < 0.001) than for radiology reports (from 100 to 93%; *p* < 0.001). Ischemic and space-occupying lesion detection was not significantly affected.

**Conclusion:**

Motion artifacts are common in suspected stroke patients, particularly in the elderly and patients with motor symptoms, reducing accuracy for hemorrhage detection by both AI and radiologists.

**Key Points:**

***Question*** *Motion artifacts reduce the quality of MRI scans, but it is unclear which factors are associated with them and how they impact diagnostic accuracy.*

***Findings*** *Motion artifacts occurred in 7% of suspected stroke MRI scans, associated with higher patient age and motor symptoms, lowering hemorrhage detection by AI and radiologists.*

***Clinical relevance*** *Motion artifacts in stroke brain MRIs significantly reduce the diagnostic accuracy of human and AI detection of intracranial hemorrhages. Elderly patients and those with motor symptoms may benefit from a greater focus on motion artifact prevention and reduction.*

**Graphical Abstract:**

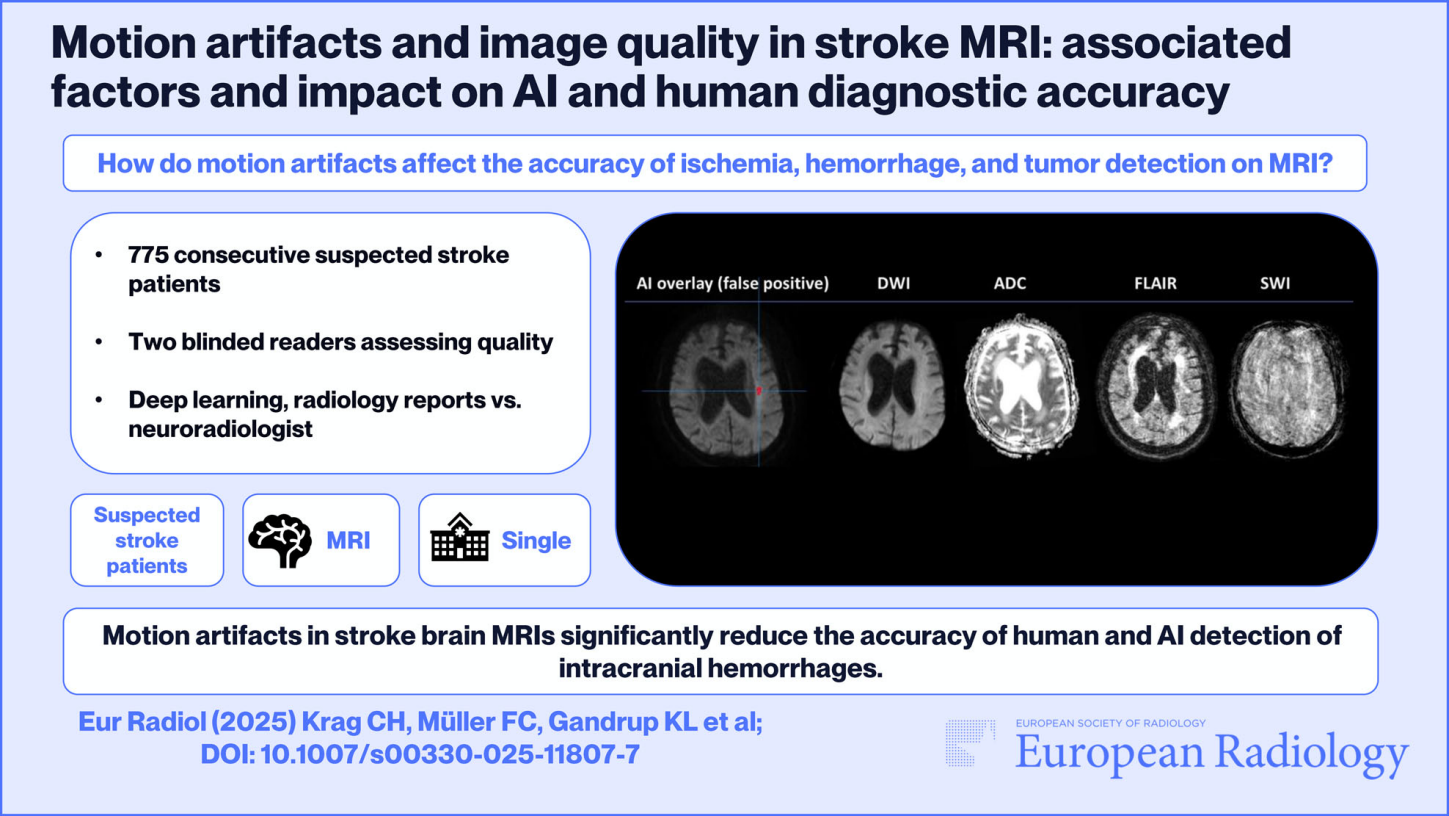

## Introduction

MRI scans are prone to motion artifacts, which decrease image quality and can lead to misinterpretation of scans [1]. Patients with clinically suspected stroke need rapid brain imaging to determine the etiology of their symptoms and to help clinicians initiate the correct treatment [2–4]. MRI scans are superior to CT scans in visualizing acute ischemic lesions [5], and for this reason, some centers have moved to an MRI-first approach for suspected stroke [6, 7]. Artificial intelligence (AI) tools detecting and classifying findings on brain MRI scans have improved in recent years, especially due to the development of convolutional neural networks [8–10]. Frequent urgent findings among suspected stroke patients include ischemic lesions, hemorrhagic lesions, and space-occupying lesions, for which several AI tools have been created to identify [11–13]. AI tools could help radiologists prioritize scans to read or clinicians to prioritize certain patients [14, 15], especially useful in high-load centers. However, previous studies have shown that deep learning tools are vulnerable to reduced image quality [16–19]. In MRI imaging, reduced image quality is often due to artifacts, some of which are inherent to the MRI techniques, such as susceptibility artifacts [20]. These can be difficult to avoid if the artifact-giving object has been operated into the patients, such as cochlear implants or aneurism coils/clips. Many MRI scans are also subject to motion artifacts due to the long sequence acquisition time. These could be limited by adjusting MRI sequence parameters, accelerating scanning acquisition with deep learning [21], or patient training or sedation [22]. However, there is limited evidence for who is at risk, and these methods may affect the image quality. There is also limited evidence on how much the motion artifacts affect human and AI diagnostic test accuracy.

Therefore, this study aimed to assess the prevalence of motion artifacts in a clinical cohort of suspected stroke patients, identify associated factors, and estimate the impact on human and AI diagnostic accuracy, as well as the proportion of AI errors attributable to motion artifacts.

## Methods

### Study design, setting, and participants

The STROBE checklist was used for manuscript preparation. The study was approved by the National Committee of Health Research in Denmark, which waived the need for informed consent (record no. 2110096). The study is a retrospective, cross-sectional study of consecutive patients from one Danish hospital scanned between January 2020 and June 2020. As a non-comprehensive stroke center, the hospital mainly receives stroke patients who are ineligible for revascularization due to contraindications or low pre-hospital triage risk, along with those requiring rehabilitation after revascularization. Inclusion criteria were: Age ≥ 18 years, brain MRI due to newly suspected stroke, maximum of 4 weeks of symptom duration before the scan. We excluded patients missing the required sequences for the AI tool (DWI, FLAIR, or SWI/T2*GRE), scans where the image quality of all sequences was too low for a reference standard, and MRI scans the AI tool could not process. We identified the MRI scans by searching the picture archiving and communications system (IMPAX 6, AGFA Healthcare). Charlson Comorbidity Index was calculated using information about the patient’s comorbidities extracted from the electronic patient journal (EPIC). The patients without severe artifacts in this cohort (*n* = 767) were previously evaluated with a deep learning tool in an external validation study [17].

### Variables

We analyzed the following variables as potential predictors for motion artifacts: age (per decade), sex (male or female), Charlson Comorbidity index (continuous), admission status (emergency department or inpatient), attempted revascularization before MRI (yes/no), patient symptoms in radiology report: decrease consciousness, visual symptoms, facial palsy, limb motor symptoms (paralysis/paresis of an extremity), ataxia, sensory symptoms (impaired or abnormal sensation), speech symptoms, dizziness (yes/no to each).

### Reference standard and radiology reports

The expert consultant neuroradiologist (K.L.G.), with 4 years of neuroradiology experience, provided the reference standard, labeling scans at the case level for acute ischemic, hemorrhagic, and space-occupying lesions. The neuroradiologist had access to the referral, and prior imaging to replicate the clinical setting. A radiology resident (C.H.K.) with 1 year of clinical radiology experience labeled the reports for the same findings. All readers, including the radiologists conducting the original report, were blinded to the AI tool’s results.

### Deep learning tool

The AI tool used in this study was the commercially available deep learning-based tool Apollo version 2.12 by Cerebriu. The tool is designed for automatic labeling and visualization of findings over 5 mm³ on brain MRIs in patients aged 15 to 75. It detects acute ischemia, hemorrhages, and intracranial tumors. Since the tool is approved for decision support, standalone testing here may be considered off-label. We did not apply upper age or lower size limits in our test population. The tool runs multiple inferences when similar sequences are sent, but when a finding is detected, it labels the entire scan, explaining our case-based analysis. The tool is approved for clinical use in the European Union (Class I—MDD). It was developed by training on 1305 internal MRI scans from 5 partner hospitals from Brazil, India, Ukraine, and the USA, from both GE, Phillips, and Siemens scanners as well as 1763 MRI scans from BraTS19 and ISLES22 datasets. Brain MRIs from our center have not been used for training the deep learning tool.

### Grading of image quality and sequences

Two radiology residents (among C.H.K., L.A., M.L.L., A.R., S.K.), independently graded DWI, FLAIR, SWI, and T2*GRE sequences using a 4-point Likert scale and noted the presence of motion artifacts. Before grading, the readers received a reference document with example ratings and artifact types, created in collaboration with an MRI research radiographer (J.M.). Likert 1 indicated non-diagnostic quality, Likert 2 adequate for diagnosis, Likert 3 minimal inhomogeneity, and Likert 4 no relevant artifacts. Usual susceptibility artifacts on DWI were specifically not assessed as it was considered inherent to that sequence. All readers were blinded to the reference standard and index test results. The average Likert score for each sequence was calculated and rounded down (e.g., 3.5 to 3). Motion and significant artifacts were recorded only if both readers agreed. For technical analysis, one reader (C.H.K.) assessed all MRI sequences for motion artifacts, as some patients had repeated sequences. We extracted relevant DICOM tags (field strength, acquisition duration, averages, acquisition type, and sequence type) using the Pydicom package in Python.

### Sensitivity analysis

We conducted two sensitivity analyses to test the robustness of our findings. In the first, repeated MRI sequences were considered markers of lower quality, reflecting radiographers’ decisions. In the second, motion artifacts were deemed present if either reader noted them.

### Statistical analysis and study size

Continuous variables are presented as mean with standard deviation (SD), while categorical variables are presented as numbers with percentages. Baseline comparisons are conducted with the Wilcoxon rank sum test (numeric variables) and χ^2^ test (categorical variables). Reader agreement was assessed with Cohen’s Kappa: κ < 0.2 (slight), 0.2–0.4 (fair), 0.4–0.6 (moderate), and > 0.6 (substantial). Multiple logistic regression was used to predict motion artifacts, with 5-fold cross-validation. Diagnostic accuracy, sensitivity, and specificity of the radiology report and AI tool were compared to the reference standard using the χ^2^ test. We examined the proportion of AI inaccurate diagnoses that are attributable to the motion artifacts, by calculating the attributable fraction of the population: ${{{\rm{AF}}}}_{{{\rm{p}}}}$=$\frac{{Ip}-{Iu}}{{Ip}}* 100 \% \,$, where I_p_ is the incidence of AI inaccuracies in the whole population, and I_u_ is the incidence of AI inaccuracies in the group not exposed to motion artifacts. The sample size of 800 consecutive unique patients balanced the need for a large external cohort, and what was feasible for the expert reader. *p*-values < 0.05 were considered statistically significant. R version 4.2.0 was used for statistical analyses.

## Results

**Fig. 1:**
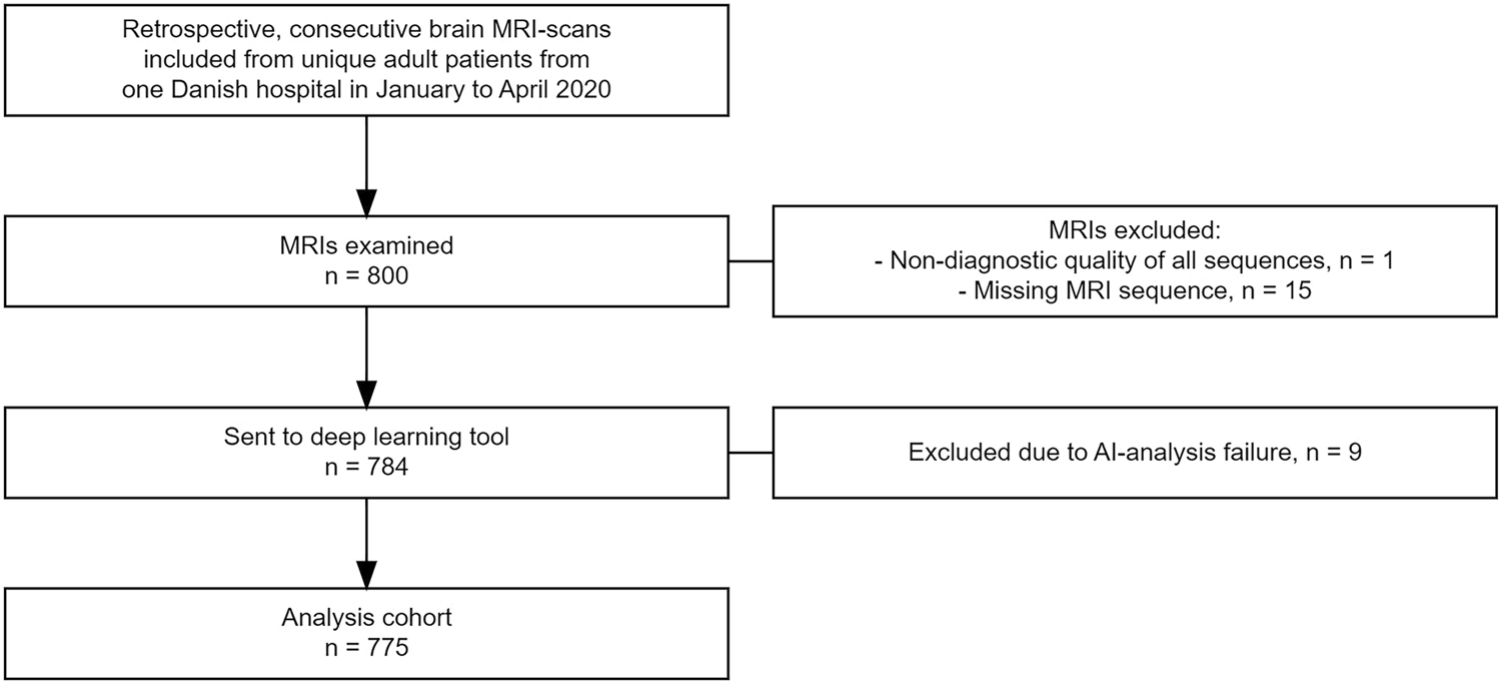
Flowchart of patient inclusion

The study included 800 consecutive patients, of which 25 were excluded due to missing key sequences (either DWI, FLAIR, or SWI/T2*GRE), non-diagnostic quality of all sequences, or due to AI-analysis failure, resulting in an analysis cohort of 775 patients (see flow diagram Fig. 1). In the analysis cohort of 775 patients, the patients had a mean age of 68 years (SD 16), 54% were female, and they had a mean Charlson Comorbidity Index score of 1.22 (SD 1.20). 76% of the patients were referred to the MRI scan from the emergency department, the remaining from in-hospital departments. We had no outpatients in the cohort. Table 1 displays key demographic and clinical characteristics.

**Table 1:** Baseline patient demographics and clinical characteristics

Variables	*N* = 775^a^
Age	68 (16)
Sex	
Female	420 (54%)
Male	355 (46%)
Charlson Comorbidity Index	1.22 (1.20)
Admission status	
Emergency department	589 (76%)
Inpatient	186 (24%)
Attempted revascularization	28 (3.6%)
MRI-symptoms (referral)	
Decreased consciousness	41 (5.3%)
Visual symptoms	68 (8.8%)
Facial palsy	66 (8.5%)
Limb motor symptoms (paresis/paralysis)	176 (23%)
Ataxia (limb/truncal/gait)	110 (14%)
Sensory symptoms	144 (19%)
Speech symptoms (aphasia/dysarthria)	137 (18%)
Dizziness	168 (22%)
MRI findings (reference)	
Acute ischemic lesions	216 (28%)
Acute hemorrhagic lesions	12 (1.5%)
Intracranial space-occupying lesions	20 (2.6%)

^a^ Mean (SD); *n* (%)

### Image quality, artifacts, and repeated sequences

**Fig. 2:**
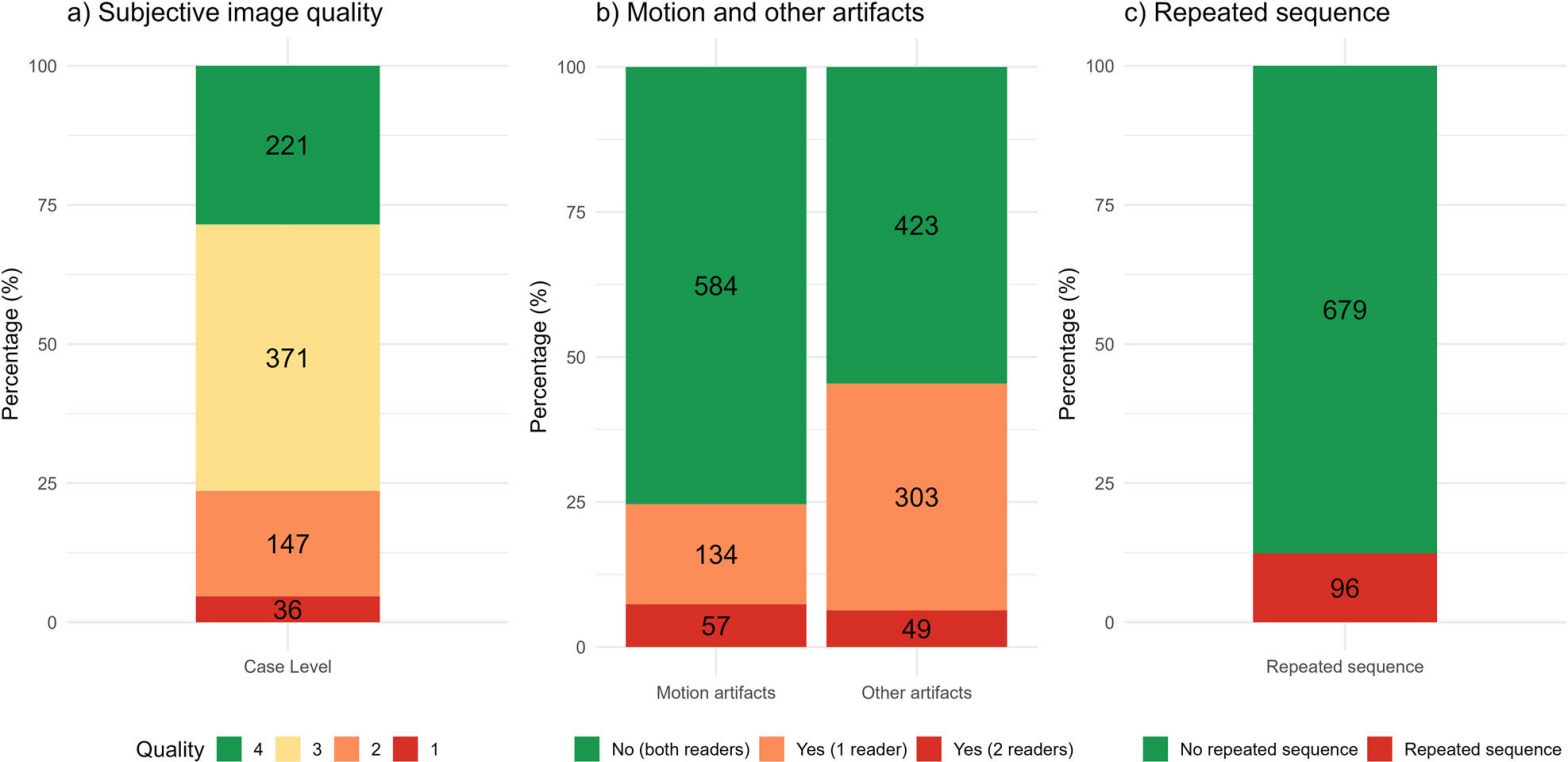
Stacked bar plots of image quality, artifacts, and repeated sequences in the analysis cohort on a case basis (*n* = 775). **a** Displays image quality on a case basis for the different MRI scans with the Likert image quality scale, by lowest quality noted on DWI/FLAIR/SWI/T2*GRE-sequences. **b** Displays the presence of motion and other artifacts on any of the DWI, FLAIR, SWI, or T2*GRE-sequences per scan. **c** Displays the scans with and without repeated DWI, FLAIR, SWI, or T2*GRE-sequences

Regarding image quality, 36 out of 775 (4.6%) scans contained at least one sequence with non-diagnostic quality (Likert 1). Motion artifacts were present on 57 of 775 scans (7.4%). 96/775 (12.4%) of the scans had repeated sequences (see Fig. 2). For the individual readers, the prevalence of motion artifacts was 15% (C.H.K.), 11% (L.A.), 25% (M.L.L.), 14% (A.R.), and 18% (S.K.).

### Inter-reader reliability

The inter-reader reliability between reader 1 and reader 2 for the grading of the different sequences ranged from slight to substantial agreement. Cohen’s Weighted Kappa for the Likert grading from 1 to 4 of DWI, FLAIR, SWI, and T2-GRE was 0.18, 0.49, 0.57, and 0.62. Cohen’s Kappa for the presence of motion artifacts on a case level was fair (0.36).

### Patient factors and motion artifacts

We found that age (mean age 67 vs. 78 years; *p* < 0.001), Charlson Comorbidity Index (mean score 1.2 vs. 1.8; *p* < 0.001), limb motor symptoms (21% vs. 46%; *p* < 0.001) to be significantly associated with the presence of motion artifacts. Meanwhile, sensory symptoms were associated with a lower occurrence of motion artifacts (19% vs. 7%; *p* = 0.020). In multivariate regression, the significance persisted for age (OR_per decade_ 1.61; *p* < 0.001), and limb motor symptoms (OR 2.36; *p* = 0.003) (see Table 2). The multivariate model had a test AUC of 0.73 (95% CI: 0.66, 0.80) for prediction of motion artifacts.

**Table 2:** Association between patient variables and motion artifacts on MRI scans

Variables	Summary statistics with baseline comparisons			Multivariate regression		
	No motion artifacts, *N* = 718^a^	Motion artifacts, *N* = 57^a^	*p*-value^b^	OR	95% CI	Multivariate *p*-value
Age (per decade)	67 (16)	78 (13)	**< 0.001**	1.61	1.26, 2.0	**< 0.001**
Sex			0.8			
Female	390 (54%)	30 (53%)				
Male	328 (46%)	27 (47%)				
Admission status			0.7			
Emergency department	547 (76%)	42 (74%)				
Inpatient	171 (24%)	15 (26%)				
Charlson Comorbidity Index	1.18 (1.18)	1.75 (1.35)	**< 0.001**	1.22	0.99, 1.49	0.06
Attempted revascularization	25 (3.5%)	3 (5.3%)	0.5			
Decreased consciousness	37 (5.2%)	4 (7.0%)	0.5			
Visual symptoms	61 (8.5%)	7 (12%)	0.3			
Facial palsy	60 (8.4%)	6 (11%)	0.6			
Limb motor symptoms	150 (21%)	26 (46%)	**< 0.001**	2.36	1.32, 4.20	**0.003**
Ataxia (limb/truncal/gait)	101 (14%)	9 (16%)	0.7			
Sensory symptoms	140 (19%)	4 (7.0%)	**0.020**	0.46	0.13, 1.19	0.2
Speech symptoms (aphasia/dysarthria)	122 (17%)	15 (26%)	0.08			
Dizziness	160 (22%)	8 (14%)	0.2			

*OR* odds ratio, *CI* confidence interval

^a^ Mean (SD); *n* (%)

^b^ Wilcoxon rank sum test; Pearson’s Chi-squared test; Fisher’s exact test, bold values indicate *p* < 0.05

### MRI-acquisition parameters

**Fig. 3:**
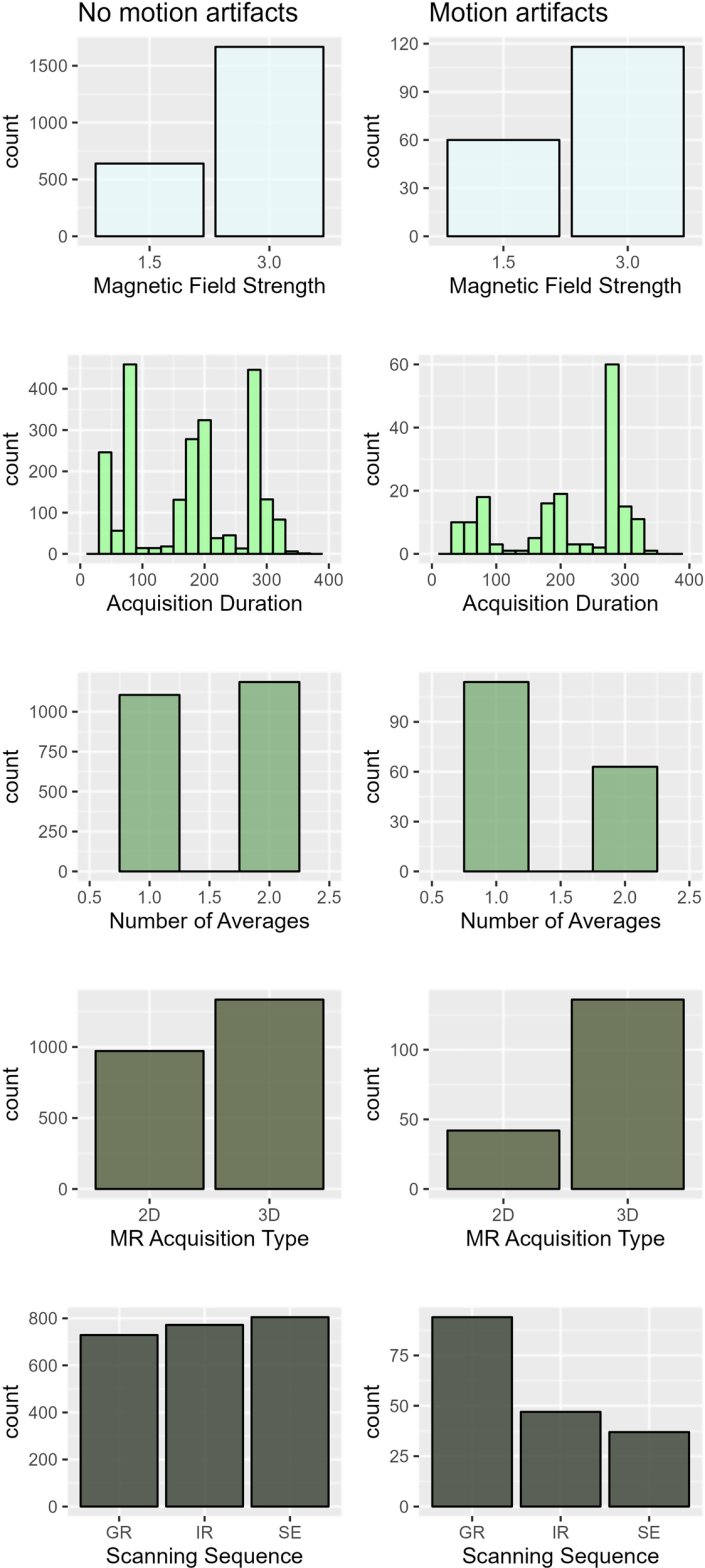
Barplots and histograms showing the distribution of MRI-acquisition parameters in sequences with no motion artifacts (left column) and sequences with motion artifacts (right column)

In the per-sequence analysis, 178 of 2484 sequences (7.2%) had motion artifacts. Longer acquisition duration, fewer averages, and 3D acquisition were significantly associated with motion artifacts. Gradient-recalled sequences (SWI and T2*GRE) were overrepresented among those with artifacts (Table 3, Fig. 3).

**Table 3:** MRI-acquisition parameters and the presence of motion artifacts

Variables	Sequences without motion artifacts, *N* = 2306^a^	Sequences with motion artifacts, *N* = 178^a^	*p*-value^b^
Magnetic field strength (Tesla)			0.09
1.5	640 (28%)	60 (34%)	
3.0	1666 (72%)	118 (66%)	
Acquisition duration (seconds)	175.0 (91.9)	209.9 (92.3)	**< 0.001**
Number of averages	1.5 (0.6)	1.4 (0.6)	**< 0.001**
MR acquisition type			**< 0.001**
2D	972 (42%)	42 (24%)	
3D	1334 (58%)	136 (76%)	
Scanning sequence			**< 0.001**
GR (gradient recalled) (SWI/T2*)	729 (32%)	94 (53%)	
IR (inversion recovery) (FLAIR)	772 (33%)	47 (26%)	
SE (spin echo) (DWI)	805 (35%)	37 (21%)	

*OR* odds ratio, *CI* confidence interval

^a^ *n* (%); Mean (SD)

^b^ Pearson’s Chi-squared test; Wilcoxon rank sum test, bold values indicate *p* < 0.05

### Diagnostic test accuracy—motion artifacts and image quality

**Fig. 4:**
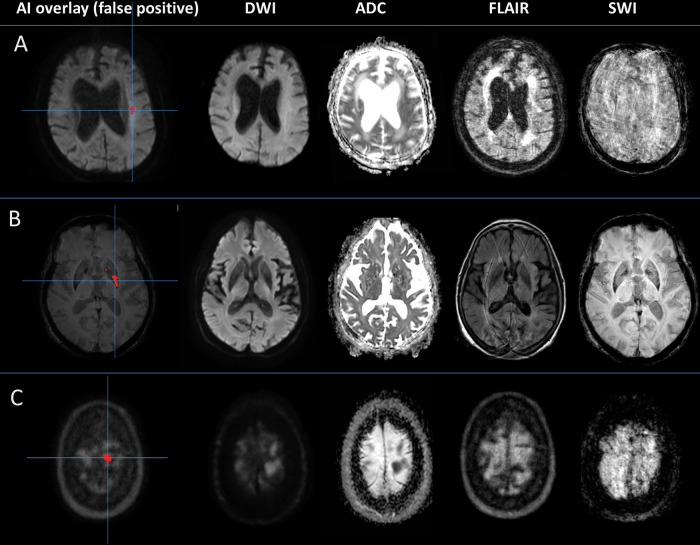
False positive predictions from AI tool in scans with motion artifacts. The left column displays the AI interface with the overlay and lesion mark. The right columns display sequences from PACS (DWI, ADC, FLAIR, and SWI). **A** 77-year-old female with decreased consciousness. Stroke MRI shows no ischemic lesions, while AI-overlay shows a false positive ischemic lesion. **B** 83-year-old female with impaired left-sided vision. Stroke MRI shows no hemorrhagic lesions, but AI-overlay shows false positive hemorrhagic lesions. **C** 74-year-old female with dysarthria and facial paresis. Stroke MRI was without space-occupying lesions, but AI-overlays show a false-positive space-occupying lesion

**Fig. 5:**
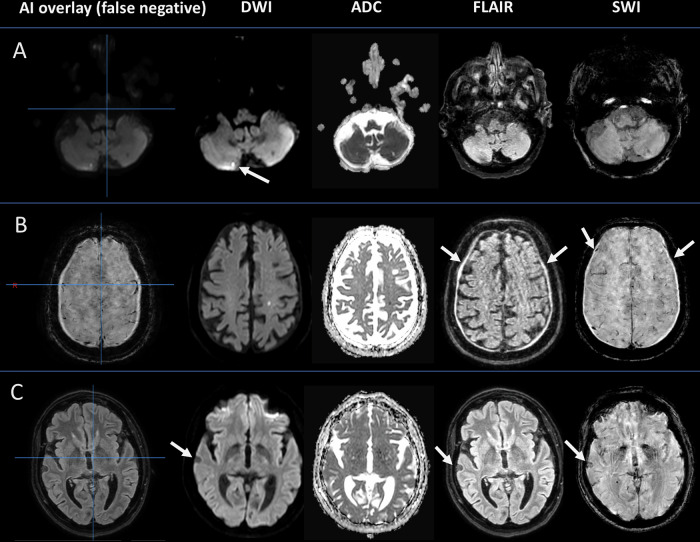
False negative predictions from AI tool in scans with motion artifacts. The left column displays the AI interface, and the right columns display sequences from PACS (DWI, ADC, FLAIR, and SWI). **A** 97-year-old male with decreased strength in the left upper extremity, facial palsy, and aphasia. Stroke MRI reveals a tiny acute ischemic lesion in the right cerebellum, overlooked by the AI tool. **B** 83-year-old male with right-sided facial palsy and right-sided hemiparesis. Stroke MRI shows bilateral hygromas/subdural hematomas, which are overlooked by the AI tool. **C** 66-year-old female with aphasia. Stroke MRI shows a tiny right-sided extra-axial tumor, not detected by the AI tool

**Fig. 6:**
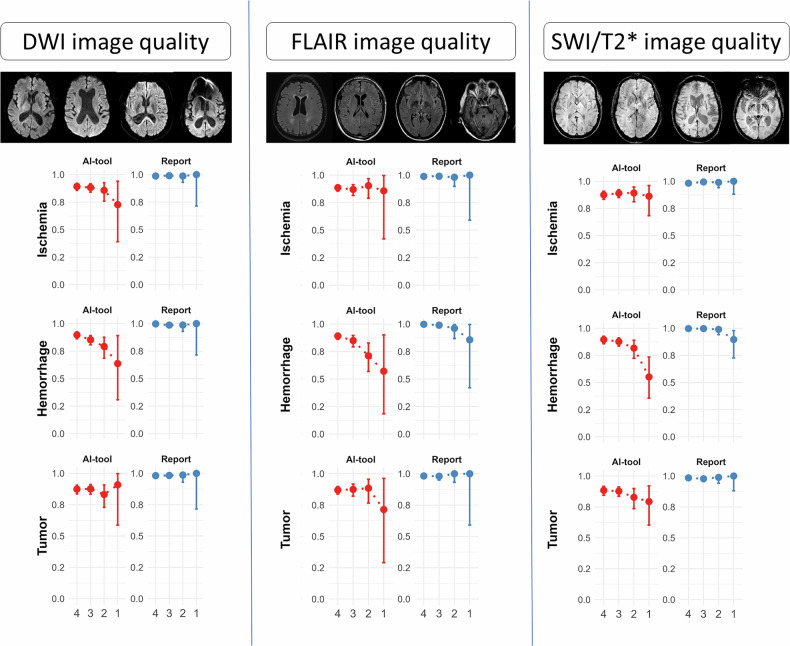
Diagnostic test accuracy of AI tool and radiology report by disease diagnosed (rows) at different sequences and image qualities (columns). The images display DWI, FLAIR, and SWI/T2* sequences, at decreasing quality (Likert 4 to the left in each column, followed by 3, 2, and 1)

Motion artifacts significantly lowered the accuracy of the AI tool for detecting hemorrhages by 21 percentage points (67% vs. 88%; *p* < 0.001). While human reader accuracy was also significantly affected, the reduction was less pronounced at a reduction of 7 percentage points (93% vs. 100%; *p* < 0.001). The accuracy of the AI tool for detecting ischemic lesions or tumor lesions was also lower in cases with motion artifacts, but not significant (see Table 4). Figure 4 displays examples of false positive cases from the AI tool, and Fig. 5 displays false negative cases. We also plotted the image quality of the different MRI sequences against the diagnostic test accuracy (Fig. 6).

**Table 4:** Diagnostic test accuracy, sensitivity, and specificity of AI tool and radiology report for detection of acute ischemia, acute hemorrhage, and tumors on brain MRI

Diagnostic accuracy^a^	Accuracy (95% CI), cases with no motion artifacts, *n* = 718	Accuracy (95% CI), cases with motion artifacts, *n* = 57	*p*-value^b^
Ischemia (AI tool)	88.6 (86.0, 90.8) [636/718]	84.2 (72.1, 92.5) [48/57]	0.29
Hemorrhage (AI tool)	88.2 (85.6, 90.4) [633/718]	66.7 (52.9, 78.6) [38/57]	**< 0.001**
Tumor (AI tool)	87.3 (84.7, 89.7) [627/718]	84.2 (72.1, 92.5) [48/57]	0.54
Ischemia (Radiology report)	98.7 (97.6, 99.4) [709/718]	100.0 (93.7, 100.0) [57/57]	1.000
Hemorrhage (Radiology report)	99.7 (99.0, 100.0) [716/718]	93.0 (83.0, 98.1) [53/57]	**< 0.001**
Tumor (Radiology report)	98.2 (96.9, 99.0) [705/718]	100.0 (93.7, 100.0) [57/57]	0.61
**Diagnostic sensitivity**^**a**^	**Sensitivity (95% CI),** **cases with no motion artifacts,** **Ischemia** ***n* = 173** **Hemorrhage** ***n* = 9** **Tumor** ***n* = 19**	**Sensitivity (95% CI),** **cases with motion artifacts,** **Ischemia** ***n* = 23** **Hemorrhage** ***n* = 3** **Tumor** ***n* = 1**	***p*-value**^**b**^
Ischemia (AI tool)	88.6 (83.3–92.7) [171/193]	91.3 (72.0–98.9) [21/23]	0.97
Hemorrhage (AI tool)	100.0 (66.4–100.0) [9/9]	66.7 (9.4–99.2) [2/3]	0.55
Tumor (AI tool)	52.6 (28.9–75.6) [10/19]	0.0 (0.0–97.5) [0/1]	1
Ischemia (Radiology report)	97.9 (94.8–99.4) [189/193]	100.0 (85.2–100.0) [23/23]	1
Hemorrhage (Radiology report)	77.8 (40.0–97.2) [7/9]	33.3 (0.8–90.6) [1/3]	0.48
Tumor (Radiology report)	78.9 (54.4–93.9) [15/19]	100.0 (2.5–100.0) [1/1]	1
**Diagnostic specificity**^**a**^	**Specificity (95% CI),** **cases with no motion artifacts,** **Ischemia** ***n* = 525** **Hemorrhage** ***n* = 709** **Tumor** ***n* = 699**	**Specificity (95% CI),** **cases with motion artifacts,** **Ischemia** ***n* = 34** **Hemorrhage** ***n* = 54** **Tumor** ***n* = 56**	***p*-value**^**b**^
Ischemia (AI tool)	88.6 (85.5–91.2) [465/525]	79.4 (62.1–91.3) [27/34]	0.19
Hemorrhage (AI tool)	88.0 (85.4–90.3) [624/709]	66.7 (52.5–78.9) [36/54]	**< 0.001**
Tumor (AI tool)	88.3 (85.6–90.6) [617/699]	85.7 (73.8–93.6) [48/56]	0.72
Ischemia (Radiology report)	99.0 (97.8–99.7) [520/525]	100.0 (89.7–100.0) [34/34]	1
Hemorrhage (Radiology report)	100.0 (99.5–100.0) [709/709]	96.3 (87.3–99.5) [52/54]	**< 0.001**
Tumor (Radiology report)	98.7 (97.6–99.4) [690/699]	100.0 (93.6–100.0) [56/56]	0.83

^a^ Diagnosis is compared to the reference standard of the neuroradiologist

^b^ Chi-square test, bold values indicate *p* < 0.05

### Attributable fraction for the population

For ischemic, hemorrhagic, and tumor detection, 10%, 18%, and 9% of inaccurate AI diagnoses occurred in cases with motion artifacts. This corresponds to an AFp of 3%, 12%, and 2%, meaning that motion artifacts accounted for these proportions of AI inaccuracies for detecting ischemia, hemorrhage, and tumors.

### Sensitivity analyses of motion artifacts

We tested the robustness of our findings by using repeated DWI, FLAIR, SWI, or T2*GRE sequences as a marker of low image quality. Higher age, comorbidity index, and limb motor symptoms were significantly associated with repeated sequences. In multivariate regression, significance remained for age and limb motor symptoms. For diagnostic test accuracy, we found non-significant decreases in accuracy for repeated sequences (AI ischemia 89% vs. 83%; *p* = 0.13, AI hemorrhage 87% vs. 82%; *p* = 0.20, AI tumor 88% vs. 82%; *p* = 0.14).

In another sensitivity analysis, motion artifacts were considered present if either reader noted them (191/775 (24.6%)). Multivariate analysis showed a significant association with higher age, comorbidities, and motor symptoms. AI and radiology report accuracy for hemorrhage detection decreased significantly in cases with motion artifacts. Meanwhile, the AFp of motion artifacts for AI hemorrhage detection was 21%, and AI tumor detection was 2%. Here we observed no decrease in AI ischemia detection (details in the electronic supplementary materials).

## Discussion

In this study, we determined the prevalence of motion artifacts on brain MRI sequences from a clinical cohort of suspected stroke patients at a large non-comprehensive stroke center. We identified patient factors and scan parameters associated with motion artifacts and estimated the impact of motion artifacts on the detection of ischemic, hemorrhagic, and structural lesions by both humans and an AI tool. Overall, we found motion artifacts in 7% of the MRI scans. SWI/T2* sequences were especially susceptible to motion artifacts, likely explained by longer acquisition duration. Our study indicated that motion artifacts were independently associated with higher patient age and motor symptoms. Motion artifacts were associated with lower diagnostic test accuracy and specificity for hemorrhage detection, and they explained up to one-fifth of the incorrect AI hemorrhage predictions. Ischemic stroke is more common, with hemorrhagic stroke accounting for only around 15% of all acute strokes [23]. While AI ischemia detection had lower specificity in cases with motion artifacts than in cases without motion artifacts, the difference was not significant. If assuming a causal relationship, motion artifacts were only able to explain a few percentages of the incorrect AI ischemic (3%) and space-occupying predictions (2%). This suggests that motion artifacts may affect some AI disease predictions more than others.

Our results also showed that the AI tool was more susceptible to motion artifacts than human readers for hemorrhage detection. Possible explanations include that the AI’s training data might have consisted mostly of higher-quality images or that human readers can better filter through the artifacts. This implies that image quality control is especially important before using diagnostic AI tools, as it can help reduce the number of false positive predictions.

Comparing the prevalence of motion artifacts to existing literature, a study of 192 MRI neuroaxis examinations found motion artifacts in 8% of outpatients and 29% of inpatient or emergency department MR examinations [24]. This indicates our two-reader definition may have been too strict, as we found motion artifacts in 7% with a two-reader definition and in 25% with a one-reader definition. However, the numbers may also reflect differences in the patient population and scanning equipment. A study on education of patients undergoing MRI for neurological conditions found motion artifacts in 16% of 316 control patients, compared to 9% of 312 in the intervention group, highlighting the effectiveness of patient education [25]. This effect may be stronger in outpatients, as some stroke patients may have difficulties comprehending instructions.

As diagnostic test accuracy studies typically exclude patients with severe artifacts, we found no comparative literature on how MRI-sequence quality affects AI and human reader accuracy. We also found no literature on associated factors for MRI motion artifacts in suspected stroke patients, though one study with head CT scans associated motion artifacts with stroke severity, older patient age, and shorter time from stroke onset [26].

Our study found that motion artifacts affected specificity more than sensitivity, a pattern also observed in deep learning models for detecting intracranial hemorrhages on non-contrast head CTs, where false positives were more common in patients with streak or motion artifacts [27]. This is also reflected in two other studies that looked at ways to reduce noise and motion artifacts to prevent false positives in fMRI data and in small target detection algorithms [28, 29].

There are several ways to deal with motion artifacts, which can be categorized into motion prevention (training, distraction, patient sedation), artifact reduction (e.g., faster imaging), and motion correction [22, 30]. Our findings of factors associated with motion artifacts hold the potential to be translated into a risk score, as we did find some prediction potential in clinical pre-MRI factors. Patients with a high predicted risk of motion artifacts could be scheduled for a specific protocol with shorter sequences and deep learning artifact reduction to minimize motion artifacts. Future studies should validate risk scores for motion artifacts in a prospective setup to improve image quality and ultimately the accuracy of diagnoses. AI developers should implement quality screening of the sequences before analyses and reject analyses of sequences with low quality [31, 32], but to be able to do that, there is an urgent need to agree on the definitions of artifacts for human readers. Automated quality assessment tools may help to quantify image quality more objectively. However, we found no tool capable of assessing DWI, FLAIR and SWI/T2* sequences for motion artifacts. While some tools exist for T1 sequences, they are not useful in this suspected stroke cohort, as T1 imaging is not included in the MRI protocol [33–35].

Some limitations apply to our study. While the sample size was sufficient for ischemia, the cohort only included a few hemorrhages and intracranial tumors. This meant we were unable to fully investigate the effect of motion artifacts on the sensitivity of detection of these lesions, which could be a subject for future studies. We found only slight agreement among readers for DWI-quality grading despite a pre-training session and distribution of grading examples. The prevalence of motion artifacts reported by the five readers varied from 11 to 25%. This suggests MRI quality evaluation is both highly subjective and sequence-dependent. It aligns with the literature, where MRI-quality-grading between readers has been described to have κ of 0.12 for grading MRI images from 1 to 5 [36]. Comprehensive stroke centers where patients have more advanced disease and symptoms may experience higher occurrences of motion artifacts. For example, we found that 11% of patients with ischemic lesions had motion artifacts, versus just 6% of patients without ischemic lesions (*p* = 0.029). While our findings of motion artifact prevalence are only generalizable to non-comprehensive stroke centers with similar patient uptake as ours, the reduced specificity in cases with motion artifacts is likely generalizable to other centers. Finally, our estimates of the radiology report diagnostic accuracy may be confounded, as the reference standard was not blinded to the report or clinical information. We aimed to ensure the highest quality reference standard for the AI comparison by including all relevant clinical data, a method also used in previous studies [37].

While our study provides new insights, future research is needed to validate and generalize the findings. Multi-center studies with diverse patient populations, different target diseases, and different AI tools would improve understanding of motion artifacts impact on diagnostic AI tools. MRI sequence developers should prioritize robustness to motion artifacts, and AI tool developers should take steps to include cases with various image quality and artifacts in their training data. Augmentation of the training data will likely reduce the susceptibility to motion artifacts we observed.

In conclusion, we found that approximately one in fourteen patients in a consecutive suspected stroke cohort have motion artifacts on their brain MRI and that it especially occurs in elderly patients, and patients with motor symptoms. SWI/T2* sequences are more susceptible to motion artifacts than DWI and FLAIR sequences. We observed significantly lower specificities for both an AI tool and human readers in detecting acute intracranial hemorrhage in cases with motion artifacts.

## Supplementary information

ELECTRONIC SUPPLEMENTARY MATERIAL

## Abbreviations

AFp: Attributable fraction for the population
AI: Artificial intelligence
DWI: Diffusion-weighted imaging
FLAIR: Fluid-attenuated inversion recovery
SWI: Susceptibility-weighted imaging
T2*GRE: T2* gradient echo
