# Supplementary material for: Motion artifacts and image quality in stroke MRI: associated factors and impact on AI and human diagnostic accuracy
Source: Eur Radiol. 2025 Jul 15;36(1):265–77. doi: 10.1007/s00330-025-11807-7 (PMC12712086; doi:10.1007/s00330-025-11807-7)
Supplement: Supplementary file 1 — ELECTRONIC SUPPLEMENTARY MATERIAL [file 330_2025_11807_MOESM1_ESM.pdf]

Motion artifacts and image quality in stroke MRI: Associated factors and impact on AI and human diagnostic accuracy

ELECTRONIC SUPPLEMENTARY MATERIAL

List of contents

Supplemental Table 1: MRI-technical parameters 2

Supplemental Table 2: Inter-reader agreement 3

Supplemental Figure 1: Sequence-specific quality on the Likert scale 4

Supplemental Table 3: Repeated sequences and patient-related variables 5

Supplemental Table 4: Motion artifacts sensitivity analysis (One of two reader definition) 6

Supplemental Table 5: Accuracy, sensitivity, and specificity by motion artifacts (one of two reader definition) 7

Supplemental Table 6: Other significant artifacts analysis (2 reader definition) 8

Supplemental Table 7: Other significant artifacts analysis (one of two reader definition) 9

Supplemental Table 8: The accuracy by the presence of other significant artifacts 10

Supplemental Table 9: Diagnostic test accuracy of AI tool and radiology report by repeated sequences. 11

Supplemental Table 10: Likert scale bi-and multivariate analysis 12

Supplemental Table 11: Scanning parameters per MRI-sequence type (DWI, FLAIR, T2*GRE, SWI) 13

Supplemental Table 12: Attributable fraction of AI errors explained by motion artifacts 14

Supplemental Table 13: Accuracy of AI findings by quality of MRI series and presence of motion artifacts 15

Supplemental Table 1: MRI-technical parameters

**Variable**	**N = 2,484**[1]
Magnetic Field Strength	
1.5	700 (28%)
3.0	1,784 (72%)
Acquisition Duration (seconds)	177.5 (92.3)
Number of Averages	1.5 (0.6)
MR Acquisition Type	
2D	1,014 (41%)
3D	1,470 (59%)
Scanning Sequence	
GR (Gradient Recalled)	823 (33%)
IR (Inversion Recovery)	819 (33%)
SE (Spin Echo)	842 (34%)
Movement artifact	
Yes	2,306 (93%)
No	178 (7.2%)

[1] n (%); Mean (SD)

Supplementary table: Baseline DICOM parameters and presence of motion artifacts on a sequence level.

Supplemental Table 2: Inter-reader agreement

	**Percentage with the same score/grading**	**Cohens Kappa**	**Cohens Weighted Kappa[1]**
DWI (Likert 1-4)	53%	0.06	0.18
FLAIR (Likert 1-4)	72%	0.24	0.49
SWI (Likert 1-4)	58%	0.28	0.57
T2-GRE (Likert 1-4)	67%	0.42	0.62
Motion artifacts (Yes/no)	83%	0.36	-
Other significant artifacts (Yes/no)	61%	0.08	-

Supplementary table

[1]Squared weight

Cohen's Kappa values for Likert-scoring of the different sequences and presence of artifacts. Color codes: red = κ < 0.2 slight agreement, yellow = κ 0.2-0.4 fair agreement, green = κ 0.4-0.6 moderate agreement, and dark green κ > 0.6 substantial agreement. Reader 1 and reader 2 display slight agreement for DWI-likert grading and the presence of other significant artifacts.

Supplemental Figure 1: Sequence-specific quality on the Likert scale

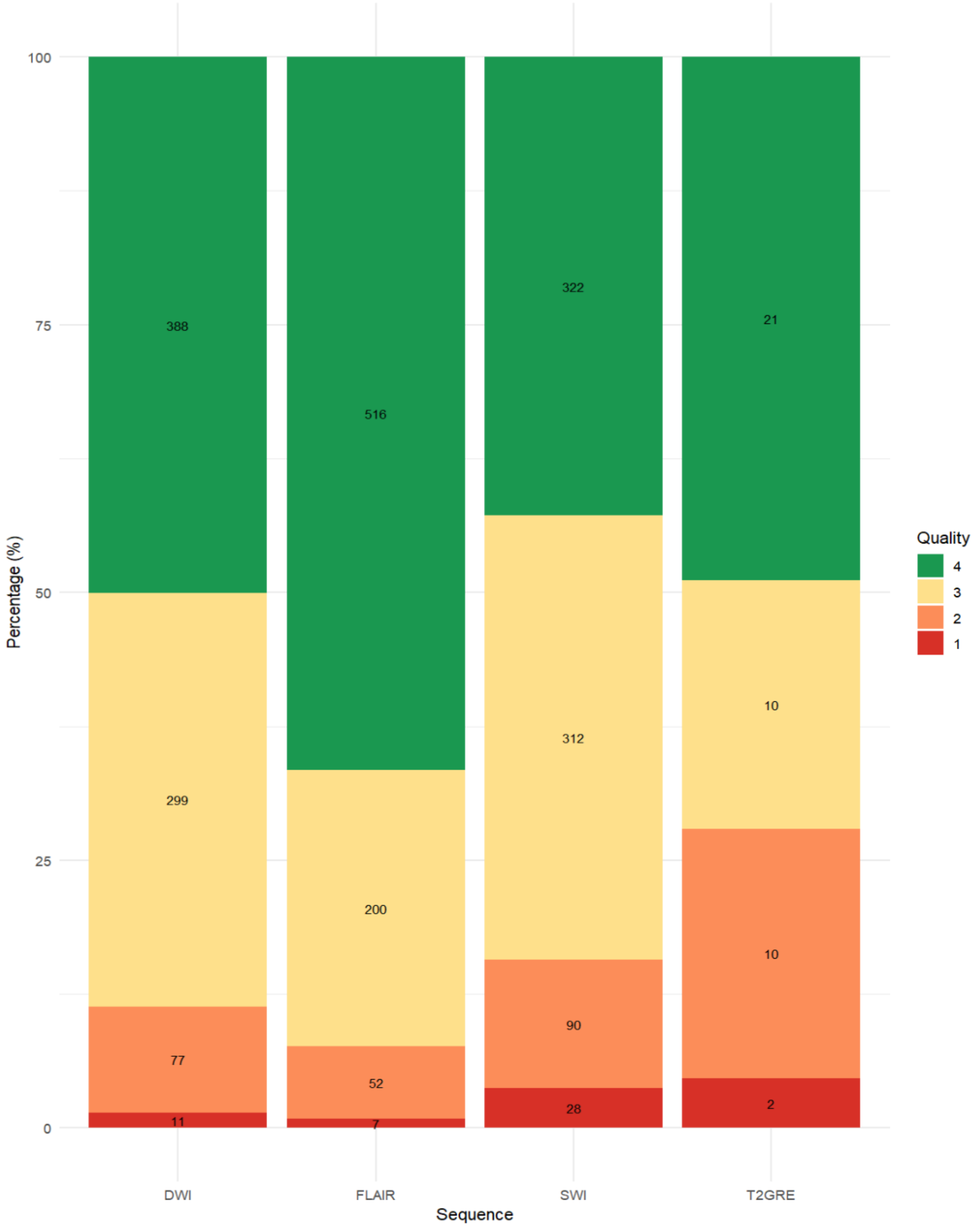

Supplemental Table 3: Repeated sequences and patient-related variables

	Summary statistics with baseline comparisons			Multivariate regression		
**Characteristic**	**No repeated MRI sequences**, N = 679[1]	**Repeated MRI-sequences**, N = 96[1]	**p-value**[2]	**OR**[3]	**95% CI**[3]	**p-value**
Age	67 (16)	74 (14)	**<0.001**	1.25	1.06, 1.49	**0.008**
Sex			0.2			
Female	362 (53%)	58 (60%)		—	—	
Male	317 (47%)	38 (40%)		0.68	0.43, 1.06	0.095
Admission status			0.076			
Emergency Department	523 (77%)	66 (69%)		—	—	
Inpatient	156 (23%)	30 (31%)		1.16	0.67, 1.95	0.6
Charlson Comorbidity Index	1.19 (1.19)	1.46 (1.27)	**0.025**	1.07	0.88, 1.27	0.5
Attempted revascularization	21 (3.1%)	7 (7.3%)	0.070	1.90	0.67, 4.92	0.2
Decreased consciousness	35 (5.2%)	6 (6.2%)	0.7	0.97	0.34, 2.33	>0.9
Visual symptoms	57 (8.4%)	11 (11%)	0.3	1.30	0.61, 2.56	0.5
Facial palsy	59 (8.7%)	7 (7.3%)	0.6	0.59	0.23, 1.31	0.2
Limb motor symptoms	141 (21%)	35 (36%)	**<0.001**	1.85	1.13, 2.99	**0.014**
Ataxia (limb/truncal/gait)	97 (14%)	13 (14%)	0.8	0.89	0.45, 1.65	0.7
Sensory symptoms	132 (19%)	12 (12%)	0.10	0.75	0.37, 1.41	0.4
Speech symptoms (afasia/dysartria)	119 (18%)	18 (19%)	0.8	0.88	0.48, 1.56	0.7
Vertigo	153 (23%)	15 (16%)	0.12	0.81	0.42, 1.49	0.5

1 Mean (SD); n (%)
2 Wilcoxon rank sum test; Pearson's Chi-squared test; Fisher's exact test
3 OR = Odds Ratio, CI = Confidence Interval

Supplementary table: Bivariate and multivariate analysis of patient factors and repeated MRI sequences.

Supplemental Table 4: Motion artifacts sensitivity analysis (One of two reader definition)

Characteristic	Summary statistics with baseline comparisons			Multivariate regression		
	**No**, N = 584[1]	**Yes**, N = 191[1]	**p-value**[2]	**OR**[3]	**95% CI**[3]	**p-value**
Age	66 (16)	74 (14)	**<0.001**	1.30	1.14, 1.48	**<0.001**
Sex			0.079			
Female	327 (56%)	93 (49%)				
Male	257 (44%)	98 (51%)				
Admission status			**0.003**			
Emergency Department	459 (79%)	130 (68%)		—	—	
Inpatient	125 (21%)	61 (32%)		1.36	0.92, 1.99	0.12
Charlson Comorbidity Index	1.12 (1.12)	1.53 (1.38)	**<0.001**	1.15	1.00, 1.32	**0.049**
Attempted revascularization	20 (3.4%)	8 (4.2%)	0.6			
Decreased consciousness	28 (4.8%)	13 (6.8%)	0.3			
Visual symptoms	52 (8.9%)	16 (8.4%)	0.8			
Facial palsy	47 (8.0%)	19 (9.9%)	0.4			
Limb motor symptoms	112 (19%)	64 (34%)	**<0.001**	1.73	1.18, 2.52	**0.005**
Ataxia (limb/truncal/gait)	81 (14%)	29 (15%)	0.7			
Sensory symptoms	122 (21%)	22 (12%)	**0.004**	0.68	0.40, 1.12	0.14
Speech symptoms (afasia/dysartria)	94 (16%)	43 (23%)	**0.044**	1.15	0.75, 1.76	0.5
Vertigo	133 (23%)	35 (18%)	0.2			

[1] Mean (SD); n (%)

[2] Wilcoxon rank sum test; Pearson's Chi-squared test

[3] OR = Odds Ratio, CI = Confidence Interval

Supplimentary table: Patient variables and presence of motion artifacts on MRI scans. The first column signifies bivariate analysis with T-test and chi-squared tests. The right column shows multivariate logistic regression of motion artifacts, including patient age, admission status, Charlson comorbidity index, limb motor symptoms, sensory symptoms and speech symptoms ((ref: the specific symptom not mentioned in referral).

Supplemental Table 5: Accuracy, sensitivity, and specificity by motion artifacts (one of two reader definition)

Diagnosis[1]	Accuracy (95% CI), cases with no motion artifacts, n = 584	Accuracy (95% CI), cases with motion artifacts, n = 191	p.value[2]
Ischemia (AI tool)	87.8 (84.9, 90.4) [513/584]	89.5 (84.3, 93.5) [171/191]	0.605
Hemorrhage (AI tool)	89.4 (86.6, 91.8) [522/584]	78.0 (71.5, 83.7) [149/191]	**<0.001**
Tumor (AI tool)	87.3 (84.4, 89.9) [510/584]	86.4 (80.7, 90.9) [165/191]	0.711
Ischemia (Radiology Report)	98.8 (97.5, 99.5) [577/584]	99.0 (96.3, 99.9) [189/191]	1.000
Hemorrhage (Radiology Report)	99.8 (99.0, 100.0) [583/584]	97.4 (94.0, 99.1) [186/191]	**0.004**
Tumor (Radiology Report)	98.1 (96.7, 99.1) [573/584]	99.0 (96.3, 99.9) [189/191]	0.745
**Diagnosis[1]**	**Sensitivity (95% CI), cases with no motion artifacts, Ischemia n = 152 Hemorrhage n = 8 Tumor n = 16**	**Sensitivity (95% CI), cases with motion artifacts, Ischemia n = 64 Hemorrhage n = 4 Tumor n = 4**	**p.value[2]**
Ischemia (AI tool)	88.8 (82.7-93.3) [135/152]	89.1 (78.8-95.5) [57/64]	1.000
Hemorrhage (AI tool)	100.0 (63.1-100.0) [8/8]	75.0 (19.4-99.4) [3/4]	0.712
Tumor (AI tool)	50.0 (24.7-75.3) [8/16]	50.0 (6.8-93.2) [2/4]	1.000
Ischemia (Radiology Report)	97.4 (93.4-99.3) [148/152]	100.0 (94.4-100.0) [64/64]	0.449
Hemorrhage (Radiology Report)	87.5 (47.3-99.7) [7/8]	25.0 (0.6-80.6) [1/4]	0.130
Tumor (Radiology Report)	75.0 (47.6-92.7) [12/16]	100.0 (39.8-100.0) [4/4]	0.675
**Diagnosis[1]**	**Specificity (95% CI), cases with no motion artifacts, Ischemia n = 432 Hemorrhage n = 576 Tumor n = 568**	**Specificity (95% CI), cases with motion artifacts, Ischemia n = 127 Hemorrhage n = 187 Tumor n = 187**	**p.value[2]**
Ischemia (AI tool)	87.5 (84.0-90.5) [378/432]	89.8 (83.1-94.4) [114/127]	0.593
Hemorrhage (AI tool)	89.2 (86.4-91.6) [514/576]	78.1 (71.5-83.8) [146/187]	**<0.001**
Tumor (AI tool)	88.4 (85.5-90.9) [502/568]	87.2 (81.5-91.6) [163/187]	0.753
Ischemia (Radiology Report)	99.3 (98.0-99.9) [429/432]	98.4 (94.4-99.8) [125/127]	0.696
Hemorrhage (Radiology Report)	100.0 (99.4-100.0) [576/576]	98.9 (96.2-99.9) [185/187]	0.096
Tumor (Radiology Report)	98.8 (97.5-99.5) [561/568]	98.9 (96.2-99.9) [185/187]	1.000

Supplementary table: Diagnostic test accuracy, sensitivity, and specificity of AI tool and radiology report for detection of acute ischemia, acute hemorrhage, and tumors on brain MRI, stratified by motion artifacts.
[1]Diagnosis is compared to the reference standard of the neuroradiologist.
[2]Chi-square test.

Supplemental Table 6: Other significant artifacts analysis (2 reader definition)

Characteristic	Summary statistics with baseline comparisons		
	**No significant other artifacts, N = 726**[1]	**Other significant artifacts, N = 49**[1]	**p-value**[2]
Age	68 (16)	67 (16)	0.7
Sex			0.9
Female	394 (54%)	26 (53%)	
Male	332 (46%)	23 (47%)	
Admission status			0.10
Emergency Department	547 (75%)	42 (86%)	
Inpatient	179 (25%)	7 (14%)	
Charlson Comorbidity Index	1.22 (1.20)	1.24 (1.32)	0.9
Attempted revascularization	28 (3.9%)	0 (0%)	0.2
Decreased consciousness	38 (5.2%)	3 (6.1%)	0.7
Visual symptoms	64 (8.8%)	4 (8.2%)	>0.9
Facial palsy	66 (9.1%)	0 (0%)	**0.017**
Limb motor symptoms	161 (22%)	15 (31%)	0.2
Ataxia (limb/truncal/gait)	103 (14%)	7 (14%)	>0.9
Sensory symptoms	134 (18%)	10 (20%)	0.7
Speech symptoms (afasia/dysartria)	129 (18%)	8 (16%)	0.8
Vertigo	158 (22%)	10 (20%)	0.8

1 Mean (SD); n (%)

2 Wilcoxon rank sum test; Pearson's Chi-squared test; Fisher's exact test

Supplementary table: Patient variables and presence of other, non-motion artifacts on MRI scans. The first column signifies bivariate analysis with T-test and chi-squared tests.

Supplemental Table 7: Other significant artifacts analysis (one of two reader definition)

Characteristic	No other significant artifacts, N = 423[1]	Other significant artifacts, N = 352[1]	p-value[2]
Age	68 (16)	68 (16)	0.6
Sex			0.2
Female	239 (57%)	181 (51%)	
Male	184 (43%)	171 (49%)	
Admission status			0.7
Emergency Department	324 (77%)	265 (75%)	
Inpatient	99 (23%)	87 (25%)	
Charlson Comorbidity Index	1.17 (1.16)	1.30 (1.24)	0.13
Attempted revascularization	14 (3.3%)	14 (4.0%)	0.6
Decreased consciousness	21 (5.0%)	20 (5.7%)	0.7
Visual symptoms	35 (8.3%)	33 (9.4%)	0.6
Facial palsy	36 (8.5%)	30 (8.5%)	>0.9
Limb motor symptoms	86 (20%)	90 (26%)	0.083
Ataxia (limb/truncal/gait)	58 (14%)	52 (15%)	0.7
Sensory symptoms	81 (19%)	63 (18%)	0.7
Speech symptoms (afasia/dysartria)	74 (17%)	63 (18%)	0.9
Vertigo	93 (22%)	75 (21%)	0.8

[1] Mean (SD); n (%)

[2] Wilcoxon rank sum test; Pearson's Chi-squared test

Supplementary table: Patient variables and presence of other, non-motion artifacts on MRI scans.

Supplemental Table 8: The accuracy by the presence of other significant artifacts

**Diagnosis[1]**	**Accuracy (95% CI), cases with no other artifacts, n = 726**	**Accuracy (95% CI), cases with other artifacts, n = 49**	**p.value[2]**
Ischemia (AI tool)	88.6 (86.0, 90.8) [643/726]	83.7 (70.3, 92.7) [41/49]	0.355
Hemorrhage (AI tool)	87.1 (84.4, 89.4) [632/726]	79.6 (65.7, 89.8) [39/49]	0.134
Tumor (AI tool)	87.9 (85.3, 90.2) [638/726]	75.5 (61.1, 86.7) [37/49]	**0.024**
Ischemia (Report)	98.8 (97.7, 99.4) [717/726]	100.0 (92.7, 100.0) [49/49]	1.000
Hemorrhage (Report)	99.2 (98.2, 99.7) [720/726]	100.0 (92.7, 100.0) [49/49]	1.000
Tumor (Report)	98.5 (97.3, 99.2) [715/726]	95.9 (86.0, 99.5) [47/49]	0.196

Supplementary table: Diagnostic test accuracy of AI tool and radiology report for detection of acute ischemia, acute hemorrhage, and tumors on brain MRI, stratified by motion artifacts.
[1]Diagnosis is compared to the reference standard of the neuroradiologist.
[2]Chi-square test.

Supplemental Table 9: Diagnostic test accuracy of AI tool and radiology report by repeated sequences.

**Diagnosis[1]**	**Accuracy (95% CI), cases with no repeated sequences, n = 679**	**Accuracy (95% CI), cases with repeated sequences, n = 96**	**p.value[2]**
Ischemia (AI tool)	89.0 (86.4, 91.2) [604/679]	83.3 (74.4, 90.2) [80/96]	0.13
Hemorrhage (AI tool)	87.2 (84.4, 89.6) [592/679]	82.3 (73.2, 89.3) [79/96]	0.20
Tumor (AI tool)	87.8 (85.1, 90.1) [596/679]	82.3 (73.2, 89.3) [79/96]	0.14
Ischemia (Radiology Report)	99.0 (97.9, 99.6) [672/679]	97.9 (92.7, 99.7) [94/96]	0.31
Hemorrhage (Radiology Report)	99.4 (98.5, 99.8) [675/679]	97.9 (92.7, 99.7) [94/96]	0.16
Tumor (Radiology Report)	98.2 (96.9, 99.1) [667/679]	99.0 (94.3, 100.0) [95/96]	1.00

Supplementary table: Diagnostic test accuracy of AI tool and radiology report for detection of acute ischemia, acute hemorrhage, and tumors on brain MRI, stratified by presence of repeated DWI/FLAIR/SWI/T2*-sequences.

[1]Diagnosis is compared to the reference standard of the neuroradiologist.

[2]Chi-square test.

Supplemental Table 10: Likert scale bi-and multivariate analysis

	Summary statistics			Multivariate regression		
**Acute ischemic lesion diagnosis**	**Inaccurate deep learning diagnosis**, N = 91[1]	**Accurate deep learning diagnosis**, N = 684[1]	**p-value**[2]	**OR**[3]	**95% CI**[3]	**p-value**
DWI	3.27 (0.80)	3.39 (0.71)	0.3	1.34	0.94, 1.89	0.10
FLAIR	3.57 (0.65)	3.58 (0.66)	0.8	0.94	0.61, 1.43	0.8
SWI/T2*GRE	3.26 (0.83)	3.23 (0.80)	0.6	0.88	0.63, 1.21	0.4

[1] Mean (SD)
[2] Wilcoxon rank sum test
[3] OR = Odds Ratio, CI = Confidence Interval

	Summary statistics			Multivariate regression		
**Acute hemorrhagic lesion diagnosis**	**Inaccurate deep learning diagnosis**, N = 104[1]	**Accurate deep learning diagnosis**, N = 671[1]	**p-value**[2]	**OR**[3]	**95% CI**[3]	**p-value**
DWI	3.15 (0.82)	3.41 (0.70)	**0.003**	1.18	0.85, 1.63	0.3
FLAIR	3.34 (0.83)	3.62 (0.62)	**<0.001**	1.28	0.89, 1.84	0.2
SWI/T2*GRE	2.92 (1.00)	3.29 (0.76)	**<0.001**	1.39	1.03, 1.86	**0.029**

[1] Mean (SD)
[2] Wilcoxon rank sum test
[3] OR = Odds Ratio, CI = Confidence Interval

	Summary statistics			Multivariate regression		
**Tumor lesion diagnosis**	**Inaccurate deep learning diagnosis**, N = 100[1]	**Accurate deep learning diagnosis**, N = 675[1]	**p-value**[2]	**OR**[3]	**95% CI**[3]	**p-value**
DWI	3.34 (0.74)	3.38 (0.72)	0.7	1.02	0.72, 1.43	>0.9
FLAIR	3.57 (0.70)	3.58 (0.65)	>0.9	0.84	0.56, 1.26	0.4
SWI/T2*GRE	3.11 (0.89)	3.26 (0.79)	0.15	1.33	0.98, 1.79	0.067

[1] Mean (SD)
[2] Wilcoxon rank sum test
[3] OR = Odds Ratio, CI = Confidence Interval

Supplementary table: AI tool accuracy by Likert scale of DWI, FLAIR, and SWI/T2*-GRE sequences with the neuroradiologist interpretation as reference.

Supplemental Table 11: Scanning parameters per MRI-sequence type (DWI, FLAIR, T2*GRE, SWI)

Characteristic	DWI, N = 842[1]	FLAIR, N = 819[1]	T2*GRE, N = 46[1]	SWI, N = 777[1]	p-value[2]
**Magnetic Field Strength**					<0.001
**1.5**	226 (27%)	230 (28%)	46 (100%)	198 (25%)	
**3.0**	616 (73%)	589 (72%)	0 (0%)	579 (75%)	
**Acquisition Duration (seconds)**	66.4 (18.7)	224.7 (48.6)	173.6 (39.6)	248.4 (59.4)	<0.001
**Number of Averages**	1.7 (0.7)	1.9 (0.3)	1.0 (0.0)	1.0 (0.0)	<0.001
**MR Acquisition Type**					<0.001
**2D**	842 (100%)	126 (15%)	46 (100%)	0 (0%)	
**3D**	0 (0%)	693 (85%)	0 (0%)	777 (100%)	
**Motion artifacts**					<0.001
**No**	805 (96%)	772 (94%)	43 (93%)	686 (88%)	
**Yes**	37 (4.4%)	47 (5.7%)	3 (6.5%)	91 (12%)	

[1] n (%); Mean (SD)

[2] Pearson's Chi-squared test; Kruskal-Wallis rank sum test

Supplemental Table 12: Attributable fraction of AI errors explained by motion artifacts

Definition of motion artifacts	Finding	Incidence of AI errors in the whole population	Incidence of AI errors in scans with no motion artifacts	AFp[1] (%)
**2 reader agreement**	Ischemic lesions	0.117	0.114	2.7
	Hemorrhagic lesions	0.134	0.118	11.8
	Space-occupying lesions	0.129	0.127	1.8
**One of two reader agreement**	Ischemic lesions	0.117	0.122	-3.5
	Hemorrhagic lesions	0.134	0.106	20.9
	Space-occupying lesions	0.129	0.127	1.8

[1]$AF_p = \frac{Ip-Iu}{Ip} * 100\%$, where I_p is the incidence of AI inaccuracies in the whole population, and I_u is the incidence of AI inaccuracies in the group without motion artifacts

Supplemental Table 13: Accuracy of AI findings by quality of MRI series and presence of motion artifacts

Ischemia AI accuracy	Motion artifacts	No motion artifacts
DWI Likert 4	89.3 (95%CI 78.1, 96.0) [50/56]	89.2 (95%CI 85.3, 92.3) [296/332]
DWI Likert 3	89.5 (95%CI 81.5, 94.8) [85/95]	87.7 (95%CI 82.4, 91.9) [179/204]
DWI Likert 2	89.2 (95%CI 74.6, 97.0) [33/37]	82.5 (95%CI 67.2, 92.7) [33/40]
DWI Likert 1	100.0 (95%CI 29.2, 100.0) [3/3]	62.5 (95%CI 24.5, 91.5) [5/8]

Hemorrhage AI accuracy	Motion artifacts	No motion artifacts
SWI Likert 4	83.3 (95%CI 51.6, 97.9) [10/12]	89.8 (95%CI 86.0, 92.9) [290/323]
SWI Likert 3	78.8 (95%CI 68.6, 86.9) [67/85]	91.0 (95%CI 86.6, 94.3) [212/233]
SWI Likert 2	83.6 (95%CI 73.0, 91.2) [61/73]	75.0 (95%CI 50.9, 91.3) [15/20]
SWI Likert 1	52.4 (95%CI 29.8, 74.3) [11/21]	62.5 (95%CI 24.5, 91.5) [5/8]

Tumor AI accuracy	Motion artifacts	No motion artifacts
FLAIR Likert 4	87.0 (95%CI 77.4, 93.6) [67/77]	87.0 (95%CI 83.5, 90.0) [382/439]
FLAIR Likert 3	86.1 (95%CI 75.9, 93.1) [62/72]	88.3 (95%CI 81.4, 93.3) [113/128]
FLAIR Likert 2	88.9 (95%CI 73.9, 96.9) [32/36]	87.5 (95%CI 61.7, 98.4) [14/16]
FLAIR Likert 1	66.7 (95%CI 22.3, 95.7) [4/6]	100.0 (95%CI 2.5, 100.0) [1/1]
